# Case report - atypical hemolytic uremic syndrome triggered by influenza B

**DOI:** 10.1186/s12882-017-0512-y

**Published:** 2017-03-20

**Authors:** Robin Kobbe, Raphael Schild, Martin Christner, Jun Oh, Sebastian Loos, Markus J. Kemper

**Affiliations:** University Medical Center Hamburg-Eppendorf, Martinistr. 52, D-20246 Hamburg, Germany

**Keywords:** Influenza, Hemolytic uremic syndrome, HUS, Atypical HUS, aHUS, Influenza vaccination, Quadrivalent

## Abstract

**Background:**

Influenza A infections have been described to cause secondary hemolytic uremic syndrome and to trigger atypical hemolytic uremic syndrome (aHUS) in individuals with an underlying genetic complement dysregulation. To date, influenza B has not been reported to trigger aHUS.

**Case presentation:**

A 6-month-old boy presented with hemolytic uremic syndrome triggered by influenza B infection. Initially the child recovered spontaneously. When he relapsed Eculizumab treatment was initiated, resulting in complete and sustained remission. A pathogenic mutation in *membrane cofactor protein (MCP)* was detected.

**Conclusion:**

Influenza B is a trigger for aHUS and might be underreported as such. Influenza vaccination may protect patients at risk.

## Background

Hemolytic uremic syndrome (HUS) is a thrombotic microangiopathic disease characterised by thrombocytopenia, hemolytical anemia, and renal impairment [1]. Most cases of HUS in industrialized countries are due to infections with pathogenic Shiga toxin-producing *Escherichia coli* (STEC) with 0157:H7 being the most common serotype, while in some tropical regions *Shigella dysenteriae type I* is the leading HUS-causing pathogen [2, 3]. Beside this HUS may be due to inborn defects of the alternative pathway regulation of the complement system, then denominated atypical HUS (aHUS). Around 10% of all HUS cases are thought to be aHUS and mutations in genes encoding for proteins in the alternative complement pathway are identified in around 50% of the patients with aHUS [4]. For aHUS an effective treatment with Eculizumab, a monoclonal anti-C5-antibody, which blocks the terminal alternative pathway activation, is available. Furthermore, non-Shiga toxin mediated causes of HUS have been associated with other infectious agents, predominantly *Streptococcus pneumoniae*, but also influenza A, HIV, and others, as well as metabolic diseases, rheumatic diseases, malignancy, pregnancy, radiation, and some drugs [4].

In most aHUS patients an ongoing active inflammatory process causes progressive damage of the nephrons leading to life-threatening complications and often to end-stage renal disease. Not all children with potential disease-causing mutations in aHUS genes develop disease and the reasons for this phenomenon, as well as the pathophysiological trigger mechanisms leading to disease manifestation are not completely understood.

Influenza A is a very common human-pathogenic virus known to directly cause secondary HUS, although historical cases reported in the literature may actually also have been due to yet undiscovered mutations in complement regulator genes. However, influenza A, predominantly pandemic H1N1 Influenza A, but also seasonal influenza A, sometimes in combination with pneumococcal co-infection, have both clearly been described to trigger aHUS [5]. These reports also included patients with aHUS after H1N1-infections who carry mutations in the gene for the complement regulator membrane cofactor protein (MCP), also known as CD46 [6]. By contrast, reports of influenza B infections triggering aHUS are missing.

## Case presentation

During the last influenza season a 6-month-old boy born to Turkish, unrelated healthy parents, was admitted after 5 days of febrile bronchitis and without any history of recent diarrhea, with pallor, edema of the eyelids and reduced urine output. Hemolytic anemia (hemoglobin 5.7 g/dL (10.1–13.1 g/dL)), thrombocytopenia (58/nL (150–500/nL)), and renal failure with raised serum creatinine (1.42 mg/dL (0.2–0.5 mg/dL)) lead to the diagnosis of HUS. A blood smear showed schistocytes, the urine analysis proteinuria and an ultrasound enlarged hyperechogenic kidneys. ADAMTS13 activity was normal, while complement activation could be confirmed by mildly reduced serum C3 (69 mg/dL (90–180 mg/dL)) and increased plasma terminal complement complex sC5b-9 (450 ng/ml (<320 ng/ml)). We could not detect any pathogenic enterobacteria (enterohemorrhagic *E. coli*, *Yersina*-, *Shigella*-, *Campylobacter* species) by culture and no Shiga toxin by PCR in multiple stool samples. A nasopharyngeal swab showed presence of Influenza B DNA by multiplex-PCR and genotyping identified the subtype Yamagata.

**Fig. 1:**
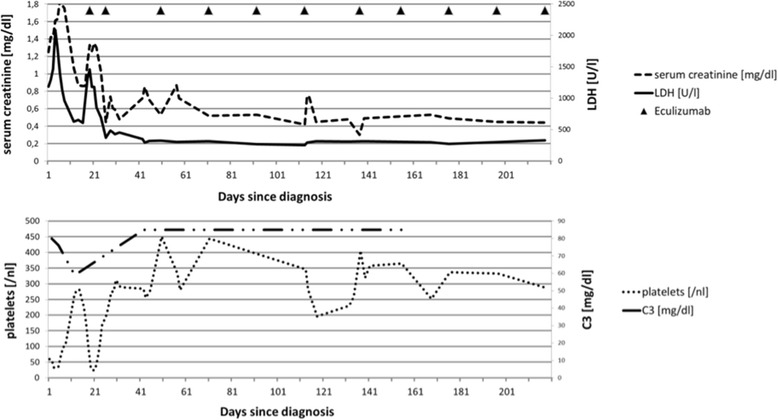
Parameters of disease activity in our patient and course of treatment with Eculizumab. LDH: lactate dehydrogenase, C3: complement component 3

Subsequently, genomic analysis of the patient’s *CFH, CFI, C3* and *MCP* genes by Sanger-sequencing identified a pathogenic heterozygous mutation in the *MCP* gene (c.104G > A (p.Cys35Tyr)) confirming the diagnosis of aHUS [7]. In addition, quantitative MLPA-analysis showed a suspicious signal of a non-allelic homologous recombination (NAHR) in the regulators of complement activation gene cluster (RCA) on chromosome 1 [8]. Meanwhile, we had started the patient on the antiviral drug Oseltamivir, antihypertensive medication and symptomatic treatment. After initial spontaneous recovery the patient developed clinical signs of relapse. Eculizumab treatment was started immediately after vaccination against meningococcal diseases. This resulted in a complete and sustained remission ever since (Fig. 1). Previously not immunized, the patient will now be vaccinated against influenza with quadrivalent vaccines for maximum protection before each start of the upcoming seasons.

## Discussion

Atypical HUS is a rare, but severe orphan disease, which occurs not in all individuals who are carrying distinctive susceptibility mutations [4]. Therefore, understanding and possibly avoiding these triggering factors is very important. We present a case of aHUS with first onset of disease following influenza B infection, putting a new pathogen on the list of triggering infectious agents. Of course it cannot be ruled out completely that disease manifestation and influenza B infection just occurred coincidentally. Furthermore, other unidentified factors might have potentially played a triggering role in our patient.

Since treatment had to be initiated while the genetic analysis was still pending we had to take decisions based on the clinical data. The age of disease onset was suggestive for aHUS. There was no diarrhea and no evidence for STEC-infection while the patient was positive for influenza B. Influenza A and *Streptococcus pneumoniae* are well known for causing secondary HUS, most likely due to viral and bacterial neuraminidases cleaving sialic acid residues from glycoproteins on erythrocytes and other surfaces of cell membranes, thereby unmasking the Thomsen-Friedenreich antigen [9–11]. In reports of influenza A triggering aHUS similar pathophysiological mechanisms have been suggested but detailed investigations or even functional analyses are not available [11–14]. Although the genomic RNA of influenza A and B viruses share structural similarities, influenza B is less common than the zoonotic influenza A virus, less genetically diverse and almost exclusively infecting humans [15]. It seems reasonable to assume that the viral neuraminidase of influenza B might have pathogenic effects similar to influenza A in triggering aHUS. Due to the relapsing course in our patient we initiated Eculizumab treatment awaiting the results of the genetic testing and found the MCP mutations described. MCP (CD46) is a cofactor for factor I, which is responsible for the inactivation of the complement components C3b and C4b, thereby protecting host cells from damage by complement [16, 17]. Patients with MCP-mutations often show a spontaneous remission, as initially in our patient, and have a lower risk to progress to end-stage renal disease than patients with other mutations in the genes for regulators for the alternative pathway complement system (e.g., factor H, factor I) [2]. Therefore, after initial weekly Eculizumab infusions, we increased the treatment intervals to every three to 4 weeks due to the stable condition of the patient. Possibly the patient could be withdrawn form Eculizumab treatment in the future.

Importantly, influenza A and B are both vaccine-preventable diseases but it remains an unanswered question if timely influenza vaccination (which included the B/Massachusetts/2/2012-like virus (Yamagata clade 2)) before disease manifestation in our patient, possibly also by vaccination of the pregnant mother, might have protected against occurrence of aHUS.

Since widespread seasonal influenza epidemics affect huge parts of the human population every year, causing substantial morbidity and mortality, pediatric infectious disease experts are calling for universal influenza vaccination for all children in Europe [18]. Protecting children by vaccination is also an effective public health strategy because children are identified as the drivers of influenza epidemics by producing high viral loads and spreading the disease to adults and high at risk populations. Most European countries already recommend influenza vaccination for any high-risk group, including children with chronic renal disease. The influenza vaccines that have been used until recently were only trivalent inactivated influenza vaccines (TIV), including split virions or highly purified H (hemagglutinin) and N (neuraminidase) particles of two different influenza A and one influenza B strains. Quadrivalent influenza vaccines include two influenza A and two influenza B strains to address the problem of influenza B mismatch. Furthermore, a highly accepted quadrivalent live attenuated influenza vaccine (LAIV) for children who do not have contraindications or precautions to this intranasal vaccine is also available. Recent analysis from Canada and the United Kingdom showed that replacement of TIV and trivalent LAIV with quadrivalent influenza vaccines might also be cost-effective [19].

Of course, only one case report of influenza B triggered aHUS might not justify the strategy mentioned above, especially as aHUS may also have occurred by another triggering event. On the other hand influenza B triggered aHUS may be underestimated and vaccination protective. The aHUS registry or other HUS databases might be able to retrospectively analyze information on safety and efficacy of influenza vaccination [20]. It might also be worth to conduct a prospective study evaluating influenza vaccination strategies in this special population to optimize care of patients with aHUS.

## Conclusion

Influenza B is a trigger for aHUS and might be underreported as such. Strategies of universal quadrivalent influenza vaccination may protect patients at risk for aHUS.

## Abbreviations

ADAMTS13: a disintegrin and metalloproteinase with a thrombo**s**pondin type 1 motif, member 13 (also known as von Willebrand factor-cleaving protease)
aHUS: atypical hemolytic uremic syndrome
*CFH*: Complement factor H
*CFI*: Complement factor I
*E. coli*: *Escherichia coli*
HIV: Human immunodeficiency virus
MCP: Membrane cofactor protein
RCA: Regulators of complement activation
STEC: Shiga toxin-producing *Escherichia coli*
